# Supplementary material for: Use of a GP-endorsed 12 months’ reminder letter to promote uptake of bowel scope screening: protocol for a randomised controlled trial in a hard-to-reach population
Source: BMJ Open. 2018 May 5;8(5):e022263. doi: 10.1136/bmjopen-2018-022263 (PMC5942414; doi:10.1136/bmjopen-2018-022263)
Supplement: Supplementary data [file bmjopen-2018-022263supp002.pdf]

ST MARK'S HOSPITAL FOUNDATION
BEATING BOWEL DISEASE. SAVING LIVES.

The North West London Hospitals NHS Trust

St. Marks' Bowel Cancer Screening Centre
St Mark's Hospital
Watford Road
Harrow
Middlesex
HA1 3UJ

**Freephone Helpline 0800 707 6060**

**<dd> <Month> <Year>**

<Title> <First Name> <Last Name>
<Address Line 1>
<Address Line 2>
<Address Line 3>
<Address Line 4>
<Postcode>

Dear <Title> <First Name> <Initial> <Last Name>,
**NHS No**: <NHS Number>

Your GP practice, WWWW WWWW WWWW WWWW WWWW WWWW WWWW WWWWW,
supports the NHS Bowel Scope Screening Programme

## Important information about your health:

We are writing to invite you for bowel scope screening, a new test available only in England that **helps prevent bowel cancer.** We last invited you for this test about a year ago.

## People aged 55+ are most at risk of bowel cancer, this test helps prevent it:

We have written to you because people who are aged 55 and over are the most at risk of developing bowel cancer. Having a Bowel Scope Screening test between the age of 55 & 59 helps prevent you from getting bowel cancer in the future. This is an important test highly recommended for **everyone** who is **55-59** years of age.

### Saving lives

The NHS offers bowel scope screening because it saves lives from bowel cancer.

Bowel scope screening is for people who **don't** have any signs of bowel cancer. The test is designed to **help prevent bowel cancer** by finding and removing small growths in the lower bowel before they turn into something more serious. These growths, called polyps, can turn into cancer over a period of years if they are left untreated. Removing these growths halves your risk of getting bowel cancer in the future.

We're lucky in Brent, Harrow, Hillingdon, and Ealing that we have the opportunity to participate in bowel scope screening. Every month about 270 people take up the test.

### What you need to do now

To book your test, simply fill in and post back the form overleaf in the Freepost envelope provided (you don't need a stamp).

We will then arrange a date and time for your bowel scope screening appointment. It takes place locally at St Mark's Hospital, which is a centre of excellence for bowel and gut medicine at Northwick Park.

Please read the enclosed leaflet, which gives more information about the test, and also has stories from people who have already been to St Mark's for bowel scope screening.

If you have any questions, please call the St Mark's Bowel Cancer Screening Centre on 020 8869 3543, or **Freephone 0800 707 60 60 to book an appointment**.

Yours sincerely,

**Sarah Marshall**
**Clinical Programme Manager, Bowel Scope Screening**

IMPORTANT – PLEASE CHECK YOUR DETAILS AND RETURN IN THE FREEPOST ENVELOPE

**Name:** <Title> <First Name> <Last Name>
**NHS Number:** <NHS Number>
**Post Code:** <Postcode>

**Please fill in your details** (either your home telephone number or your mobile number is required; this is so we can contact you to confirm your appointment)**:**

Home number: ______________________

Mobile number: ______________________

(You may receive the details of your appointment in a text message reminder a week before your appointment)

Please **tick** this box if you would like to have a bowel scope screening appointment:

☐ I'd like to arrange a bowel scope screening appointment at St Mark's Hospital in Harrow.

Please **tick** as appropriate: My preferred appointment time(s) would be:

| | **Afternoon 13:00-15:30** | **Evening 16:45-19:00** |
|---|---|---|
| **Monday** | | |
| **Tuesday** | | |
| **Wednesday** | | |
| **Thursday** | | |
| **Friday** | | |

When we receive your form, we'll contact you with a suggested date and time for your appointment.

**(Tear here)**
