# Supplementary material for: Use of a GP-endorsed 12 months’ reminder letter to promote uptake of bowel scope screening: protocol for a randomised controlled trial in a hard-to-reach population
Source: BMJ Open. 2018 May 5;8(5):e022263. doi: 10.1136/bmjopen-2018-022263 (PMC5942414; doi:10.1136/bmjopen-2018-022263)
Supplement: Author's manuscript [file bmjopen-2018-022263.draft_revisions.pdf]

BMJ Open is committed to open peer review. As part of this commitment we make the peer review history of every article we publish publicly available.

When an article is published we post the peer reviewers' comments and the authors' responses online. We also post the versions of the paper that were used during peer review. These are the versions that the peer review comments apply to.

The versions of the paper that follow are the versions that were submitted during the peer review process. They are not the versions of record or the final published versions. They should not be cited or distributed as the published version of this manuscript.

BMJ Open is an open access journal and the full, final, typeset and author-corrected version of record of the manuscript is available on our site with no access controls, subscription charges or pay-per-view fees (http://bmjopen.bmj.com).

If you have any questions on BMJ Open's open peer review process please email info.bmjopen@bmj.com

BMJ Open

Use of a GP-endorsed 12 months' reminder letter to promote uptake of bowel scope screening: Protocol for a randomised controlled trial in a hard-to-reach population.

Journal:	*BMJ Open*
Manuscript ID	bmjopen-2018-022263
Article Type:	Protocol
Date Submitted by the Author:	08-Feb-2018

Complete List of Authors:	von Wagner, Christian; UCL, Research Department of Behavioural Science and Health<br>Hirst, Yasemin; University College London, Research Department of Behavioural Science and Health<br>Tookey, Sara; UCL, Research Department of Behavioural Science and Health<br>Kerrison, Robert; University College London, Research Department of Behavioural Science and Health<br>Marshall, Sarah; London North West Healthcare NHS Trust, St Mark's Bowel Cancer Screening Centre<br>Prentice, Andrew; London North West Healthcare NHS Trust, St Mark's Bowel Cancer Screening Centre<br>Vulkan, Daniel; Queen Mary University of London, Policy Research Unit in Cancer Awareness, Screening and Early Diagnosis, Wolfson Institute of Preventive Medicine, Barts and The London School of Medicine and Dentistry<br>Macleod, Una; Hull York Medical School<br>duffy, stephen; Queen Mary University of London, 3Policy Research Unit in Cancer Awareness, Screening and Early Diagnosis, Wolfson Institute of Preventive Medicine, Barts and The London School of Medicine and Dentistry

Keywords:	Colorectal Cancer, Flexible Sigmoidoscopy, GP endorsement, Reminders

SCHOLARONE™
Manuscripts

Use of a GP-endorsed 12 months' reminder letter to promote uptake of bowel scope screening: Protocol for a randomised controlled trial in a hard-to-reach population.

Running Title: GP-endorsed reminders for bowel scope screening

Authors: von Wagner, Christian [1]; Hirst, Yasemin, Tookey, Sara [1]; Kerrison, Robert [1]; Marshall, Sarah[2]; Prentice, Andrew [2]; Vulkan, Daniel [3]; MacLeod, Una [4]; Stephen Duffy[3]

Affiliations: [1]Research Department of Behavioural Science and Health, University College London, Gower Place, London, UK

[2]St Mark's Bowel Cancer Screening Centre, London North West Healthcare NHS Trust, Harrow, United Kingdom

[3]Policy Research Unit in Cancer Awareness, Screening and Early Diagnosis, Wolfson Institute of Preventive Medicine, Barts and The London School of Medicine and Dentistry, Queen Mary University of London, London, UK

[4]Centre for Health and Population Sciences, Hull York Medical School, University of York, Heslington, York, UK

Corresponding Author: Dr Christian von Wagner, Research Department of Behavioural Science and Health, University College London, London WC1E6BT, UK; E-mail: c.wagner@ucl.ac.uk; Phone: +44 (0)20 7679 1614

Abstract word count: 296 ; **Main text word count:**;2699 **Figures:** 1 **Tables:** 0; Appendices: A, B

Keywords: Colorectal Cancer Screening; Flexible Sigmoidoscopy, GP-endorsement, reminders, London.

For peer review only

For peer review only - http://bmjopen.bmj.com/site/about/guidelines.xhtml

Abstract

Introduction: Flexible sigmoidoscopy (FS) screening is associated with reduced CRC incidence and mortality when offered as a one-off test to men and women aged 55-64. The test, also referred to as the 'bowel scope screening' (BSS) test, was added to England's national Bowel Cancer Screening Programme (BCSP) in March 2013, where it is offered to men and women aged 55. Since its implementation, uptake of the BSS test has been low, with only 43% of the eligible population attending an appointment. Sending non-participants a reminder at age 56 has been shown to improve uptake by up to nine percentage points at a single centre in London; we hypothesise that adding a General Practitioners (GPs) endorsement to the reminder could improve uptake even further.

Methods and analysis: This paper describes the protocol for a randomised controlled trial, which will examine the effectiveness of adding a GPs endorsement to a reminder for BSS non-participants aged 56. All screening-eligible adults who have not responded to a BSS appointment at London North West Healthcare NHS Trust within 12 months' of their initial invitation will be randomised to receive either a GP-endorsed reminder letter or reminder letter without GP endorsement. The primary outcome will be the proportion of individuals screened within each group eight weeks after the reminder. Statistical comparisons will be made using univariate and multivariate logistic regression, with 'uptake' as the outcome variable, GP reminder group as the exposure and socio-demographic variables as covariates.

Ethics and dissemination: The study was approved by the Yorkshire & Humber - Bradford Leeds Research Ethics Committee (16/YH/0298) and the Confidentiality Advisory Group (17/CAG/0162). The results will be disseminated in a peer-reviewed journal in accordance with the Consort statement and will be made available to the public.

Trial Registration: Current Controlled Trials ISRCTN 82867861

For peer review only - http://bmjopen.bmj.com/site/about/guidelines.xhtml

Strengths and limitations of the study

- If effective, this study will provide a cost-effective means to promote uptake of FS screening in an organised programme.
- The key importance of this randomised controlled trial protocol is that it highlights a methodology for delivering GP endorsed reminders to examine whether a 12 months' reminder letter can be further enhanced to improve screening uptake in an organised FS-based screening programme for CRC.
- There is a strong public health mandate to develop system friendly interventions to promote uptake of the BSS programme in England, particularly among socioeconomically deprived groups, where the potential for improving uptake is greatest.
- The study population is limited to the patients who are registered at the participating GP practices in London Boroughs of Hillingdon, Harrow and Brent.
- The coverage of GP endorsement will be limited to those areas served by GPs willing to participate in our trial.

For peer review only

For peer review only - http://bmjopen.bmj.com/site/about/guidelines.xhtml

Introduction

Colorectal cancer (CRC) is the fourth most common cancer in the United Kingdom and the second leading cause of cancer deaths [1; 2]. Screening is able to improve survival by detecting CRC early, when it is more treatable [3; 4; 5; 6]. Screening can additionally prevent cases through the early detection and removal of adenomas: the precancerous lesions from which most CRCs develop [7; 8; 9].

Randomised controlled trials (RCTs) examining the effectiveness of flexible sigmoidoscopy (FS) screening to improve CRC outcomes highlight that disease-specific mortality and incidence are reduced by 32% and 50% (respectively) when the test is performed just once between the ages of 55 and 64 [8; 10; 11; 12]. On this basis, the National Health Service (NHS) in England added 'once-only' FS screening for men and women aged 55 (known as Bowel Scope Screening, BSS) to the National Bowel Cancer Screening Programme (BCSP) in March 2013 [13]. Since that time, uptake of FS in the English BCSP has been only 43% [14], which is considerably lower than the uptake for the faecal occult blood test, which is offered biennially to those aged 60-74, and dramatically lower than rates observed for breast and cervical screening (54%, 76%, 78% respectively, [15; 16; 17]).

Barriers to FS screening include practical barriers (i.e., inconvenient appointment time, difficulties travelling to the appointment and so on), worry about pain, discomfort, or injury associated with the examination, and not wanting to know about any health issues [18]. Several studies attempting to address these barriers have examined the use of self-referral reminders (reminders which prompt former non-participants to self-refer for screening) 12 and 24 months after their initial invitation [19-21]. These have been shown to facilitate uptake in as much as 21.5% of former non-participants. While these results are highly promising, the annual reminder concept is still relatively unexplored, and there is considerable scope for modification and refinement of the intervention content. For example, there is now considerable evidence that a General Practitioners (GPs) endorsement of

For peer review only - http://bmjopen.bmj.com/site/about/guidelines.xhtml

cancer screening is positively associated with uptake [22- 24.]. Previous research examining the relative importance of barriers to CRC screening indicate that primary care should play a key role in encouraging uptake, highlighting that the two most important barriers to CRC screening among individuals who have never been screened are 'failure of the clinician to suggest screening' and 'not knowing testing was necessary' [25].

A recent study conducted in Australia demonstrated that a theory-based modification to the advanced notification letter improved uptake among men and was highly cost-effective [22]. A recent review has found that the inclusion of a GP's endorsement on the invitation letter can improve uptake of CRC screening with the faecal occult blood test (FOBt); however, none of the studies identified in the review used flexible sigmoidoscopy screening uptake as an outcome [26]. This paper describes the protocol for a RCT which will examine the effectiveness of adding a GP endorsement to a reminder sent to BSS non-responders 12 months after their initial invitation.

Aims

The primary aim of this RCT will be to test whether adding a GP endorsement to the 12 months' reminder letter improves the uptake of BSS among previous non-responders over and above a 12 months reminder letter without a GP endorsement. The secondary aim will be to examine demographic differences in uptake in response to the GP-endorsed reminder letter and the standard annual reminder letter (i.e. a reminder without a GP endorsement).

Methods and analysis

Study Design

This study will be a non-clinical RCT with two parallel arms. The intervention group will receive a GP-endorsed reminder letter (Appendix A) by post, the control group will receive the same reminder letter by post, minus the GP-endorsement (Appendix B).

For peer review only - http://bmjopen.bmj.com/site/about/guidelines.xhtml

Study Setting

This RCT will be conducted in London, England, at London North West Healthcare NHS Trust in Harrow in between April and July, 2018.

Eligibility Criteria

Adults will be eligible to take part in the study if they: 1) are aged 56 years at the time of enrolment, 2) are registered with a consenting general practice served by the BSS centre at London North West Healthcare NHS Trust ; 3) have been offered, but not responded to a routine BSS appointment for at least twelve months; 4) meet the clinical eligibility criteria for BSS and 5) have not opted out from of sharing their personal data for purposes beyond direct care (from here on referred to as Type 2 objectors).

NHS Digital, formerly known as the Health and Social Care Information Centre, will be responsible for identifying potentially eligible adults and excluding any individuals who do not meet the criteria for the study. NHS Digital will exclude individuals if they: 1) have an open episode status, e.g. postponed the appointment to another date; 2) contacted the screening centre and declined; 3) contacted the screening programme and were deemed medically unfit; 4) contacted the screening programme to confirm they would be attending their appointment, but then did not attend.

Blinding & Randomisation

Adults who are eligible for inclusion will be entered into a computerised study database and randomised (in a 1:1 ratio) to receive either a ‘standard reminder letter’ or a ‘GP-endorsed reminder letter’ 12 months’ after their initial invitation using simple pseudo-random allocation methods.

Pre-intervention Phase: Recruitment of Practices

For peer review only - http://bmjopen.bmj.com/site/about/guidelines.xhtml

We will send invitations to GP practices which are being served by London North West Healthcare NHS Trust and have been included in the BSS programme for at least 12 months. Consenting GP practices will be permitting their practice name to be included in the GP-endorsed reminder letter. Using a recruitment strategy similar to a recent randomised controlled trial with GP practices in London, we are expecting approximately 50% of GP practices to sign up using a strategy of email invitations and weekly reminders [24].

Intervention Phase: Reminder delivery

The 12 months' reminder letter (Standard Reminder Group)

The standard reminder letter will be the same reminder letter that was used in the previous trials (see Appendix A ; [19-21]). It will be a personally addressed letter from BSS centre at the North West London Hospitals Trust that will invite recipients to make an appointment by returning an 'appointment-request-slip' or calling the Freephone number at the screening centre. As with the previous trials, the reminder will also give recipients the option to express a preference for the day and time of their appointment.

The 12 months' reminder letter with GP-Endorsement (Intervention Group)

The GP-endorsed reminder letter will be the same as the standard reminder letter, except that it will contain an additional statement of GP-endorsement, which states: "Your GP practice, xxxx xxxxx, supports this NHS Bowel Scope Screening Programme" (see Appendix B).

- Figure 1 Here –

Timeline

Twelve months after receiving an initial invitation for BSS, all eligible adults will receive a reminder letter (either the endorsed or standard letter) with the option of indicating preferred days and times to schedule their BSS appointment at the screening centre based at London North West Healthcare NHS Trust.

For peer review only - http://bmjopen.bmj.com/site/about/guidelines.xhtml

Sample size calculation

The study has been designed to detect a 5 percentage point increase in uptake between the GP-endorsed and standard reminder group. As a GP-endorsed reminder has not previously been tested in the context of an organised FS screening programme for CRC, the estimates for this study were based on the effect size of using GP-endorsement to promote uptake of screening for CRC using alternative tests (such as the faecal occult blood test: [22-24]). Hewitson and colleagues' [24] found that the response rate of individuals receiving usual care, versus a GP endorsed invitation letter, increased the rate of response by 5%, from 10% (as observed for a standard reminder in previous work) to 15%. To detect this increase in uptake with 80% power at the 5% alpha level, with two sided testing, approximately 600 participants per trial arm are required, giving a total sample size of 1,200 participants.

Data processing and data collection

1) Identification: Adults will be identified by NHS Digital. Identification of eligible participants will take place using data contained within the Bowel Cancer Screening System (BCSS), which provides an up-to-date electronic record of uptake data for individuals enrolled in the national BSS.
2) Data Cleanse: NHS Digital will query the data of non-participants to identify type-II opt-outs. Using the cleansed dataset, the first 1200 individuals will be selected to be included in the RCT database.
3) Randomisation: NHS Digital will randomise eligible participants using the pre-randomised dataset. The database will be split into two separate files with an equal number of people in each document using the study groups.
4) Intervention: NHS Digital will share the data associated with each mail-out for the intervention and the control group with the mailing company called CFH Docmail Limited. CFH Docmail is an NHS Information Governance toolkit accredited mailing company that will be facilitating the mail merge throughout the study. The dataset will include study group, unique ID, personal contact details, NHS number and the

For peer review only - http://bmjopen.bmj.com/site/about/guidelines.xhtml

practice name (only for intervention group). Reminders will be produced by merging each study group database with the reminder letter templates. The full package will include: the reminder letter, standard information booklet and a freepost return envelope. This process of sending previous non-participants a reminder 12 months' after their initial will continue until the study sample size (n=1,200) is reached. We plan to send out 150 letters per week and send out all the reminders in eight weeks.

5) Data Generation: Once letters are sent, we will allow eight weeks for all individual's respond to the self-referral reminder. At the 16th week of the trial, NHS Digital will query the screening episode status of all individuals using their NHS numbers. If previous non-responders did not respond, their status will not change and they will remain 'non-responders'. If people confirmed and attended their appointment, they will be coded as 'attended'. If people contacted the centre and cancelled their appointment and did not book another appointment, they will be considered 'decliners'. If someone confirmed an appointment but did not attend, they will be coded as 'non-attenders'. We will also request a separate category for those who have contacted the screening centre, had the initial confirmation and attended the clinic but not eligible to have the full investigation. People will be adequately screened, if they have had the full bowel scope investigation and received a definitive clinical result. For this purpose, we will additionally request the screening outcome of those who had the screening, and also information about the follow-up colonoscopy investigations. As a result, we will be able to distinguish between: 1) those who reacted to the self-referral reminders (responders) vs. those who did not (non-responders), 2) those who successfully attended screening (attenders) vs. those who did not (non-attenders), and 3) those who were adequately screened vs. those who were not. In addition, NHS Digital will provide the Index of Multiple Deprivation (IMD) scores, using the postcodes of each individual included in the study.

For peer review only - http://bmjopen.bmj.com/site/about/guidelines.xhtml
For peer review only

6) Data Extraction: At the end of the study, after all data has been collated, the study database will be duplicated by NHS Digital. One copy will be anonymised for analysis by the UCL research team. In the anonymisation process, all identifiable information including NHS number, full name, Address, GP code, and GP name will be excluded.
7) Data Deletion: Once the anonymised dataset is sent to the research team at UCL and verified, the research team will ask CFH Docmail to destroy all the datasets that were shared by NHS Digital.

Primary outcome

The primary outcome of this study will be the proportion of individuals attending a BSS appointment within each group. Uptake will be determined by checking the episode status of each individual included in the study 8 weeks after the distribution of the final reminder letter.

Statistical Analysis

The primary outcome will be analysed to test for a significant difference in uptake between the two groups using univariate and multivariate logistic regression, with 'adequately screened' as the outcome measure, 'trial arm' as the exposure and 'gender' and IMD as the covariates. Secondary analyses will look at whether there are differences in sociodemographic characteristics of non-responders, decliners and non-attenders. Sample characteristics will be reported using descriptive statistics. The comparison of overall uptake between trial arms will be presented using odds ratios (ORs) and 95% confidence intervals (CIs).

Ethics and dissemination

The timeliness and the feasibility of the study was approved by the Public Health England Bowel Cancer Screening Programme Research Advisory Committee (ID_192). The ethical considerations were reviewed by the Yorkshire & Humber - Bradford Leeds Research Ethics Committee (16/YH/0298), who provided a favourable opinion. The study was also submitted

For peer review only - http://bmjopen.bmj.com/site/about/guidelines.xhtml

to the Confidentiality Advisory Group (17/CAG/0162) for Section 251 exemption of the National Health Service Act 2006, which permits individual data to be processed without consent when the reasons for no consent is justified and the proposed study is in the public interest. However, the project website is designed to inform members of the public who will be included in the trial, timelines, objectives and how their data will be processed (http://www.ucl.ac.uk/iehc/research/behavioural-science-health/research/cancer-communication-screening/gp-reminders-for-bss-non-participants). As part of this patient and public notification, individuals are given the opportunity to opt-out in line with the CAG requirements. The explicit consent was not deemed necessary because the annual reminders are part being sent as routine practice at the BSS centre at London North West Healthcare NHS Trust as part of a Commissioning for Quality and Innovation (CQUIN) awarded by NHS England in 2017, and at the end of the project, the research team at UCL will not be receiving identifiable information. The exemption was necessary for the secure and fair data processing between NHS Digital and CFH Docmail. A Section 251 exemption was permitted to the CFH Docmail to receive the name, address, and the NHS number of the non-responders of BSS from NHS Digital for the duration of the study and subsequently post the annual reminders.

We will disseminate the outcomes of the study using academic publications, the study website, social media and also send a report to the GP practices that were invited to the study once the results are published. We will aim to publish the results in a peer-reviewed journal in accordance with the CONSORT statement. We will also use the study website to inform the public about the study outcomes: http://www.ucl.ac.uk/iehc/research/behavioural-science-health/research/cancer-communication-screening/gp-reminders-for-bss-non-participants.

Trial Registration

This trial has been registered with the International Standard Randomised Controlled Trial Number Registry (ISRCTN: 82867861).

For peer review only - http://bmjopen.bmj.com/site/about/guidelines.xhtml

Contributors

CVW and RK conceived of the study. ST, YH, CVW and RK participated in its design. ST, YH, CVW, RK and ST wrote the first draft of the manuscript. RK and YH participated in the power calculations and statistical analysis. DV, AP, SM, SD were involved in revisions of the manuscript. All authors read and approved the final manuscript.

Acknowledgements

We would like to acknowledge Josephine Ruwende and Jean-Pierre Laake for their advice and assistance in the recruitment of GPs for this study.

Funding

This work is part of the programme of the Policy Research Unit in Cancer Awareness, Screening, and Early Diagnosis, which receives funding for a research programme from the Department of Health Policy Research Programme (106/001). The funder has played play no role in study design and will have no influence in data collection, management, analysis and interpretation or any decisions about whether and where to publish the results of the study.

It is a collaboration between researchers from seven institutions (Queen Mary University of London, University College London, King's College London, London School of Hygiene and Tropical Medicine, Hull York Medical School, Durham University and Peninsula Medical School).

Competing interests

The authors declare that they have no competing interests.

Provenance and peer review

For peer review only - http://bmjopen.bmj.com/site/about/guidelines.xhtml

For peer review only

Not commissioned; externally peer reviewed

Data sharing statement

The data that support the findings of this study are available from the Bowel Cancer Research Committee, but restrictions apply to the availability of these data, which were used under license for the current study, and so are not publicly available. Anonymised data are can be made available from the authors upon reasonable request and with permission of the Bowel Cancer Research Advisory Committee after an application is made for to the Office for Data Release for a data sharing agreement.

For peer review only

For peer review only - http://bmjopen.bmj.com/site/about/guidelines.xhtml

References:

1. Cancer Research UK: Bowel Cancer Incidence Statistics. 2016a. from: http://www.cancerresearchuk.org/health-professional/cancer-statistics/statistics-by-cancer-type/bowel-cancer/incidence. (Accessed 22 Sept 2016).

2. Cancer Research UK: Bowel Cancer Mortality Statistics. 2016b. From: http://www.cancerresearchuk.org/health-professional/cancer-statistics/statistics-by-cancer-type/bowel-cancer/mortality. (Accessed 22 Sept 2016.)

3. Cancer Research UK: 2016c. from: http://www.cancerresearchuk.org/health-professional/cancer-statistics/statistics-by-cancer-type/bowel-cancer/survival. (Accessed 22 Sept 2016).

4. Sung J, Ng S, Chan F, Chiu H, Kim H, Matsuda T, Ng S, Lau J, Zheng S, Adler S. An updated Asia Pacific Consensus Recommendations on colorectal cancer screening. *Gut* 2015, 64(1):121-132.

5. Council of the European Union: Council Recommendation of 2 December 2003 on cancer screening. *Off J Eur Union* 2003; 878/EC: 34–38.

6. Logan RF, Patnick J, Nickerson C, Coleman L, Rutter MD, von Wagner C: Outcomes of the Bowel Cancer Screening Programme (BCSP) in England after the first 1 million tests. *Gut* 2012, 61(10):1439-1446. doi: 10.1136/gutjnl-2011-300843

7. Leslie A, Carey FA, Pratt NR, Steele RJC. The colorectal adenoma–carcinoma sequence. *Br J Surg* 2002, 89(7): 845-860. doi: 10.1046/j.1365-2168.2002.02120.x

For peer review only - http://bmjopen.bmj.com/site/about/guidelines.xhtml

8. Atkin WS, Edwards R, Kralj-Hans I, Wooldrage K, Hart AR, Northover JM, Parkin DM, Wardle J, Duffy SW, Cuzick J. Once-only flexible sigmoidoscopy screening in prevention of colorectal cancer: a multicentre randomised controlled trial. *Lancet* 2010, 375(9726): 1624-1633. doi: 10.1016/S0140-6736(10)60551-X.

9. Whyte S, Chilcott J, Halloran S. Reappraisal of the options for colorectal cancer screening in England. *Colorectal Dis* 2012, 14(9): e547-e561. doi: 10.1111/j.1463-1318.2012.03014.x

10. Hoff Geir, Grotmol Tom, Skovlund Eva, Bretthauer Michael. Risk of colorectal cancer seven years after flexible sigmoidoscopy screening: randomised controlled trial. *BMJ* 2009, 338 :b1846. doi: https://doi.org/10.1136/bmj.b1846

11. Schoen R., Pinsky P., Weissfeld J., Yokochi L., Church T., Laiyemo A., et al. Colorectal-cancer incidence and mortality with screening flexible sigmoidoscopy. *N Engl J Med*, 2012, 366: 2345-2357. doi: 10.1056/NEJMoa1114635

12. Segnan N., Armaroli P., Bonelli L., Risio M., Sciallero S., Zappa M., et al. Once-only sigmoidoscopy in colorectal cancer screening: follow-up findings of the Italian Randomized Controlled Trial–SCORE.2011. *J Natl Cancer Inst* 2001, 103: 1310–1322. doi: 10.1093/jnci/djr284

13. Department of Health NHS bowel cancer screening programme: Piloting of flexible sigmoidoscopy (FS) – Advice to the NHS and bidding process. 2012. from: https://www.gov.uk/government/publications/piloting-of-flexible-sigmoidoscopy-advice-to-the-nhs-and-bidding-procress (Accessed 23 Sept 2015).

For peer review only

For peer review only - http://bmjopen.bmj.com/site/about/guidelines.xhtml

14. McGregor LM, Bonello B, Kerrison RS, Nickerson C, Baio G, Berkman L, Rees CJ, Atkin W, Wardle J, von Wagner C. Uptake of Bowel Scope (Flexible Sigmoidoscopy) Screening in the English National Programme: the first 14 months'. *J Med Screen* 2015; e-pub ahead of print 20 September 2015. doi: 10.1177/0969141315604659

15. Von Wagner C, Baio G, Raine R, Snowball J, Morris S, Atkin W, et al. Inequalities in participation in an organized national colorectal cancer screening programme: results from the first 2.6 million invitations in England. *Int J Epidemiol* 2011;40(3):712–8. doi: 10.1093/ije/dyr008

16. Health & Social Care Information Centre (HSCIC). Breast screening Programme, England – 2014-15 [NS]. 2016. From: http://content.digital.nhs.uk/catalogue/PUB20018. (Accessed 10 Oct 2016).

17. Health & Social Care Information Centre (HSCIC). Cervical screening Programme, England – 2014-15 [NS]. 2016. http://content.digital.nhs.uk/article/2021/Website-Search?productid=19239&q=cervical+screening&sort=Relevance&size=10&page=1&area=both#top. (Accessed 10 Oct 2016).

18. Vernon SW. Participation in colorectal cancer screening: a review. *J Natl Cancer Inst* 1997, 89(19): 1406–1422.

19. Kerrison RS, McGregor LM, Marshall S, Isitt J, Counsell N, Wardle J, von Wagner C. Use of a 12 months'' self-referral reminder to facilitate uptake of bowel scope (flexible sigmoidoscopy) screening in previous non-responders: a London-based feasibility study. *British journal of cancer,* 2016 114(7):751-758. doi: https://doi.org/10.1093/jnci/89.19.1406

For peer review only - http://bmjopen.bmj.com/site/about/guidelines.xhtml

20. Kerrison, RS, McGregor, LM, Marshal, S, Isitt, J, Counsell, N, Rees, CJ, von Wagner, C. Improving uptake of flexible sigmoidoscopy screening: a Randomised Controlled Trial of non-participant reminders in the English Screening Programme. *Endoscopy* 2017, 49, 39-43. doi: 10.1055/s-0042-118452

21. Kerrison, R. S., McGregor, L. M., Counsell, N., Marshall, S., Prentice, A., Isitt, J., Rees, C. J. and von Wagner, C. (2018) 'Use of Two Self-referral Reminders and a Theory-Based Leaflet to Increase the Uptake of Flexible Sigmoidoscopy in the English Bowel Scope Screening Program: Results From a Randomized Controlled Trial in London', *Annals of Behavioral Medicine*. doi: 10.1093/abm/kax068.

22. Zajac IT, Duncan AC, Flight I, Wittert GA, Cole SR, Young GP, Wilson CJ, Turnbull DA. Theory-based modifications of an advanced notification letter improves screening for bowel cancer in men: A randomised controlled trial. Social Science & Medicine. 2016;165:1-9. doi: 10.1016/j.socscimed.2016.06.036

23. Raine R, Duffy SW, Wardle J, Solmi F, Morris S, Howe R, Kralj-Hans I, Snowball J, Counsell N, Moss S, Hackshaw A. Impact of general practice endorsement on the social gradient in uptake in bowel cancer screening. British journal of cancer. 2016 Feb;114(3):321.

24. Hewitson P, Ward AM, Heneghan C, Halloran SP, Mant D. Primary care endorsement letter and a patient leaflet to improve participation in colorectal cancer screening: results of a factorial randomised trial. *Br J Cancer* 2011, 105(4): 475-480. doi: 10.1038/bjc.2011.255

25. Jones, R. M., Woolf, S. H., Cunningham, T. D., Johnson, R. E., Krist, A. H., Rothemich, S. F. and Vernon, S. W. (2010) 'The Relative Importance of Patient-

For peer review only - http://bmjopen.bmj.com/site/about/guidelines.xhtml

Reported Barriers to Colorectal Cancer Screening', *American Journal of Preventive Medicine*. Elsevier, 38(5), pp. 499–507. doi: 10.1016/J.AMEPRE.2010.01.020.

26. Senore C, Inadomi J, Segnan N, Bellisario C, Hassan C: Optimising colorectal cancer screening acceptance: a review. Gut 2015: gutjnl-2014-308081. doi: 10.1136/gutjnl-2014-308081.

27. Hirst, Y., Skrobanski, H., Kerrison, R. S., Kobayashi, L. C., Counsell, N., Djedovic, N., Ruwende, J., Stewart, M. and von Wagner, C. ; Text-message Reminders in Colorectal Cancer Screening (TRICCS): a randomised controlled trial', *British Journal of Cancer* 2017; 116(11):1408-1414. doi: 10.1038/bjc.2017.117.

For peer review only

For peer review only - http://bmjopen.bmj.com/site/about/guidelines.xhtml

Figure 1: GP-Endorsed Reminders Consort Flow diagram

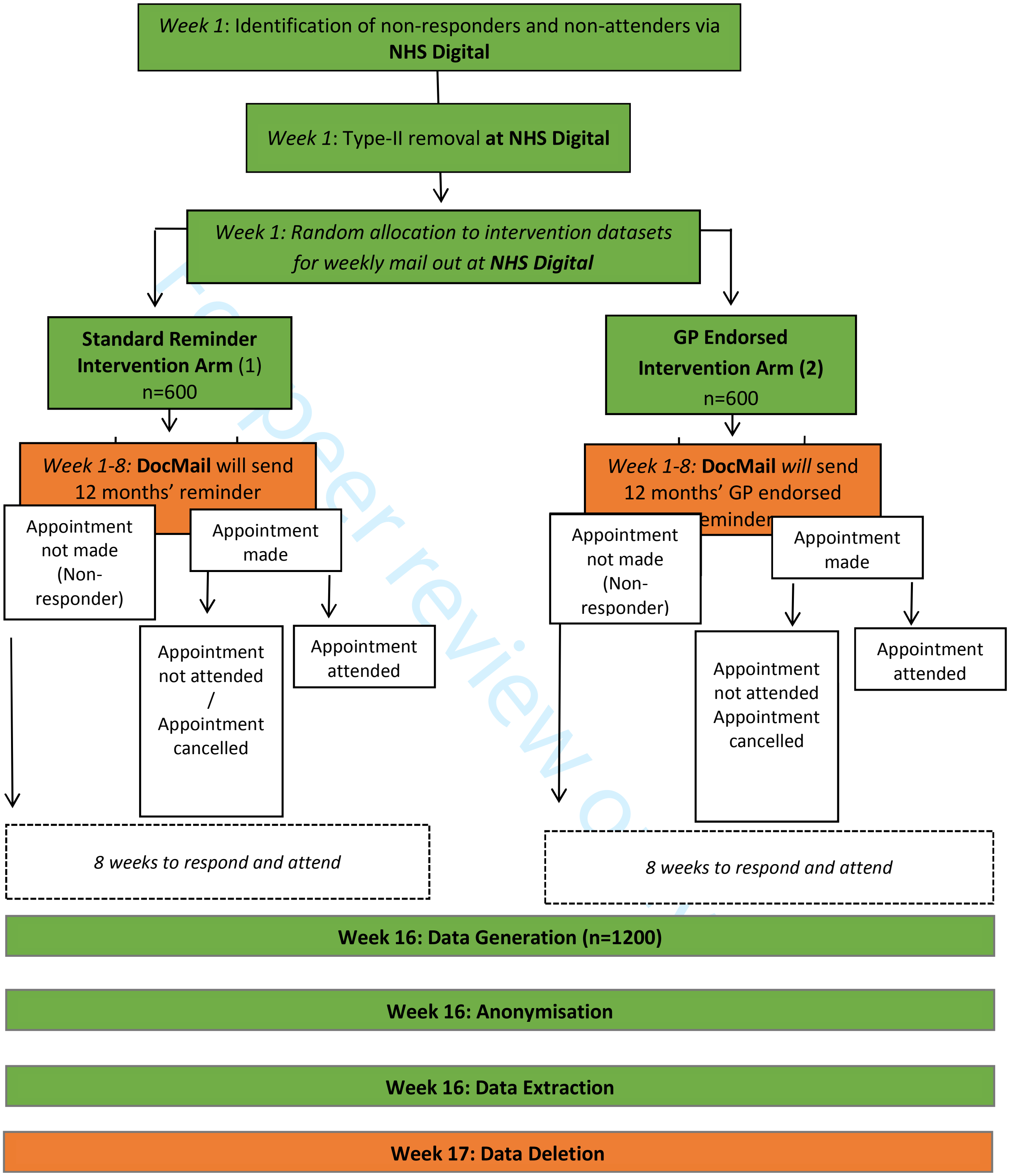

2

For peer review only

The North West London Hospitals NHS Trust

St. Marks' Bowel Cancer Screening Centre
St Mark's Hospital
Watford Road
Harrow
Middlesex
HA1 3UJ

Freephone Helpline 0800 707 6060

<dd> <Month> <Year>

<Title> <First Name> <Last Name>
<Address Line 1>
<Address Line 2>
<Address Line 3>
<Address Line 4>
<Postcode>

Dear <Title> <First Name> <Initial> <Last Name>,
NHS No: <NHS Number>

Important information about your health:

We are writing to invite you for bowel scope screening, a new test available only in England that **helps prevent bowel cancer.** We last invited you for this test about a year ago.

People aged 55+ are most at risk of bowel cancer, this test helps prevent it:

We have written to you because people who are aged 55 and over are the most at risk of developing bowel cancer. Having a Bowel Scope Screening test between the age of 55 & 59 helps prevent you from getting bowel cancer in the future. This is an important test highly recommended for **everyone** who is **55-59** years of age.

Saving lives
The NHS offers bowel scope screening because it saves lives from bowel cancer.

Bowel scope screening is for people who **don't** have any signs of bowel cancer. The test is designed to **help prevent bowel cancer** by finding and removing small growths in the lower bowel before they turn into something more serious. These growths, called polyps, can turn into cancer over a period of years if they are left untreated. Removing these growths halves your risk of getting bowel cancer in the future.

We're lucky in Brent, Harrow, Hillingdon and Ealing that we have the opportunity to participate in bowel scope screening. Every month about 270 people take up the test.

What you need to do now
To book your test, simply fill in and post back the form overleaf in the Freepost envelope provided (you don't need a stamp).

We will then arrange a date and time for your bowel scope screening appointment. It takes place locally at St Mark's Hospital, which is a centre of excellence for bowel and gut medicine at Northwick Park.

Please read the enclosed leaflet, which gives more information about the test, and also has stories from people who have already been to St Mark's for bowel scope screening.

If you have any questions, please call the St Mark's Bowel Cancer Screening Centre on 020 8869 3543, or **Freephone 0800 707 60 60 to book an appointment**.

Yours sincerely,

Sarah Marshall
Clinical Programme Manager, Bowel Scope Screening

For peer review only

For peer review only - http://bmjopen.bmj.com/site/about/guidelines.xhtml

ST MARK'S HOSPITAL FOUNDATION
BEATING BOWEL DISEASE. SAVING LIVES.

The North West London Hospitals NHS Trust NHS

IMPORTANT – PLEASE CHECK YOUR DETAILS AND RETURN IN THE FREEPOST ENVELOPE

Name: <Title> <First Name> <Last Name>
NHS Number: <NHS Number>
Post Code: <Postcode>

Please fill in your details (either your home telephone number or your mobile number is required; this is so we can contact you to confirm your appointment)**:**

Home number: ______________________

Mobile number: ______________________

(You may receive the details of your appointment in a text message reminder a week before your appointment)

Please **tick** this box if you would like to have a bowel scope screening appointment:

☐ I'd like to arrange a bowel scope screening appointment at St Mark's Hospital in Harrow.

Please **tick** as appropriate: My preferred appointment time(s) would be:

	**Afternoon 13:00-15:30**	**Evening 16:45-19:00**
**Monday**		
**Tuesday**		
**Wednesday**		
**Thursday**		
**Friday**		

When we receive your form, we'll contact you with a suggested date and time for your appointment.

(Tear here)

For peer review only - http://bmjopen.bmj.com/site/about/guidelines.xhtml

The North West London Hospitals NHS Trust

St. Marks' Bowel Cancer Screening Centre
St Mark's Hospital
Watford Road
Harrow
Middlesex
HA1 3UJ

Freephone Helpline 0800 707 6060

<dd> <Month> <Year>

<Title> <First Name> <Last Name>
<Address Line 1>
<Address Line 2>
<Address Line 3>
<Address Line 4>
<Postcode>

Dear <Title> <First Name> <Initial> <Last Name>,
NHS No: <NHS Number>

Your GP practice, WWWW WWWW WWWW WWWW WWWW WWWW WWWW WWWWW,
supports the NHS Bowel Scope Screening Programme

Important information about your health:

We are writing to invite you for bowel scope screening, a new test available only in England that **helps prevent bowel cancer.** We last invited you for this test about a year ago.

People aged 55+ are most at risk of bowel cancer, this test helps prevent it:

We have written to you because people who are aged 55 and over are the most at risk of developing bowel cancer. Having a Bowel Scope Screening test between the age of 55 & 59 helps prevent you from getting bowel cancer in the future. This is an important test highly recommended for **everyone** who is **55-59** years of age.

Saving lives
The NHS offers bowel scope screening because it saves lives from bowel cancer.

Bowel scope screening is for people who **don't** have any signs of bowel cancer. The test is designed to **help prevent bowel cancer** by finding and removing small growths in the lower bowel before they turn into something more serious. These growths, called polyps, can turn into cancer over a period of years if they are left untreated. Removing these growths halves your risk of getting bowel cancer in the future.

We're lucky in Brent, Harrow, Hillingdon, and Ealing that we have the opportunity to participate in bowel scope screening. Every month about 270 people take up the test.

What you need to do now
To book your test, simply fill in and post back the form overleaf in the Freepost envelope provided (you don't need a stamp).

We will then arrange a date and time for your bowel scope screening appointment. It takes place locally at St Mark's Hospital, which is a centre of excellence for bowel and gut medicine at Northwick Park.

Please read the enclosed leaflet, which gives more information about the test, and also has stories from people who have already been to St Mark's for bowel scope screening.

If you have any questions, please call the St Mark's Bowel Cancer Screening Centre on 020 8869 3543, or **Freephone 0800 707 60 60 to book an appointment**.

For peer review only - http://bmjopen.bmj.com/site/about/guidelines.xhtml

The North West London Hospitals NHS Trust

Yours sincerely,

Sarah Marshall
Clinical Programme Manager, Bowel Scope Screening

IMPORTANT – PLEASE CHECK YOUR DETAILS AND RETURN IN THE FREEPOST ENVELOPE

Name: <Title> <First Name> <Last Name>
NHS Number: <NHS Number>
Post Code: <Postcode>

Please fill in your details (either your home telephone number or your mobile number is required; this is so we can contact you to confirm your appointment)**:**

Home number: ________________________

Mobile number: _______________________

(You may receive the details of your appointment in a text message reminder a week before your appointment)

Please **tick** this box if you would like to have a bowel scope screening appointment:

☐ I'd like to arrange a bowel scope screening appointment at St Mark's Hospital in Harrow.

Please **tick** as appropriate: My preferred appointment time(s) would be:

	**Afternoon 13:00-15:30**	**Evening 16:45-19:00**
**Monday**		
**Tuesday**		
**Wednesday**		
**Thursday**		
**Friday**		

When we receive your form, we'll contact you with a suggested date and time for your appointment.

(Tear here)

GP endorsed 12 month's reminder letter, version 1.2, 18/07/2017, **IRAS ID: 200928** 2/2

For peer review only

STANDARD PROTOCOL ITEMS: RECOMMENDATIONS FOR INTERVENTIONAL TRIALS

SPIRIT 2013 Checklist: Recommended items to address in a clinical trial protocol and related documents*

Section/item	ItemNo	Description	Page Numbers
**Administrative information**			
Title	1	Descriptive title identifying the study design, population, interventions, and, if applicable, trial acronym	Page 1
Trial registration	2a	Trial identifier and registry name. If not yet registered, name of intended registry	Page 2
	2b	All items from the World Health Organization Trial Registration Data Set	N/A
Protocol version	3	Date and version identifier	Footer
Funding	4	Sources and types of financial, material, and other support	Page 12
Roles and responsibilities	5a	Names, affiliations, and roles of protocol contributors	Page 12
	5b	Name and contact information for the trial sponsor	Page 12.
	5c	Role of study sponsor and funders, if any, in study design; collection, management, analysis, and interpretation of data; writing of the report; and the decision to submit the report for publication, including whether they will have ultimate authority over any of these activities	Page 12

For peer review only

	5d	Composition, roles, and responsibilities of the coordinating centre, steering committee, endpoint adjudication committee, data management team, and other individuals or groups overseeing the trial, if applicable (see Item 21a for data monitoring committee)	Page 12
**Introduction**			
Background and rationale	6a	Description of research question and justification for undertaking the trial, including summary of relevant studies (published and unpublished) examining benefits and harms for each intervention	Page 4-5
	6b	Explanation for choice of comparators	
Objectives	7	Specific objectives or hypotheses	Page 5
Trial design	8	Description of trial design including type of trial (eg, parallel group, crossover, factorial, single group), allocation ratio, and framework (eg, superiority, equivalence, noninferiority, exploratory)	Page 6-10
**Methods: Participants, interventions, and outcomes**			
Study setting	9	Description of study settings (eg, community clinic, academic hospital) and list of countries where data will be collected. Reference to where list of study sites can be obtained	Page 6

For peer review only

Eligibility criteria	10	Inclusion and exclusion criteria for participants. If applicable, eligibility criteria for study centres and individuals who will perform the interventions (eg, surgeons, psychotherapists)	Page 6
Interventions	11a	Interventions for each group with sufficient detail to allow replication, including how and when they will be administered	Page 7
	11b	Criteria for discontinuing or modifying allocated interventions for a given trial participant (eg, drug dose change in response to harms, participant request, or improving/worsening disease)	N/A
	11c	Strategies to improve adherence to intervention protocols, and any procedures for monitoring adherence (eg, drug tablet return, laboratory tests)	N/A
	11d	Relevant concomitant care and interventions that are permitted or prohibited during the trial	N/A
Outcomes	12	Primary, secondary, and other outcomes, including the specific measurement variable (eg, systolic blood pressure), analysis metric (eg, change from baseline, final value, time to event), method of aggregation (eg, median, proportion), and time point for each outcome. Explanation of the clinical relevance of chosen efficacy and harm outcomes is strongly recommended	Page 10

For peer review only - http://bmjopen.bmj.com/site/about/guidelines.xhtml

Participant timeline	13	Time schedule of enrolment, interventions (including any run-ins and washouts), assessments, and visits for participants. A schematic diagram is highly recommended (see Figure)	Page 7
Page 7Sample size	14	Estimated number of participants needed to achieve study objectives and how it was determined, including clinical and statistical assumptions supporting any sample size calculations	Page 8
Recruitment	15	Strategies for achieving adequate participant enrolment to reach target sample size	Page8-9

Methods: Assignment of interventions (for controlled trials)

Allocation:

Sequence generation	16a	Method of generating the allocation sequence (eg, computer-generated random numbers), and list of any factors for stratification. To reduce predictability of a random sequence, details of any planned restriction (eg, blocking) should be provided in a separate document that is unavailable to those who enrol participants or assign interventions	Page 8
Allocation concealment mechanism	16b	Mechanism of implementing the allocation sequence (eg, central telephone; sequentially numbered, opaque, sealed envelopes), describing any steps to conceal the sequence until interventions are assigned	Page 8

Implementation	16c	Who will generate the allocation sequence, who will enrol participants, and who will assign participants to interventions	Page 8-9
Blinding (masking)	17a	Who will be blinded after assignment to interventions (eg, trial participants, care providers, outcome assessors, data analysts), and how	
	17b	If blinded, circumstances under which unblinding is permissible, and procedure for revealing a participant's allocated intervention during the trial	
**Methods: Data collection, management, and analysis**			
Data collection methods	18a	Plans for assessment and collection of outcome, baseline, and other trial data, including any related processes to promote data quality (eg, duplicate measurements, training of assessors) and a description of study instruments (eg, questionnaires, laboratory tests) along with their reliability and validity, if known. Reference to where data collection forms can be found, if not in the protocol	Page 8-10
	18b	Plans to promote participant retention and complete follow-up, including list of any outcome data to be collected for participants who discontinue or deviate from intervention protocols	

Data management	19	Plans for data entry, coding, security, and storage, including any related processes to promote data quality (eg, double data entry; range checks for data values). Reference to where details of data management procedures can be found, if not in the protocol	Page 8-10
Statistical methods	20a	Statistical methods for analysing primary and secondary outcomes. Reference to where other details of the statistical analysis plan can be found, if not in the protocol	Page 10
	20b	Methods for any additional analyses (eg, subgroup and adjusted analyses)	Page 10
	20c	Definition of analysis population relating to protocol non-adherence (eg, as randomised analysis), and any statistical methods to handle missing data (eg, multiple imputation)	N/A
**Methods: Monitoring**			
Data monitoring	21a	Composition of data monitoring committee (DMC); summary of its role and reporting structure; statement of whether it is independent from the sponsor and competing interests; and reference to where further details about its charter can be found, if not in the protocol. Alternatively, an explanation of why a DMC is not needed	N/A

	21b	Description of any interim analyses and stopping guidelines, including who will have access to these interim results and make the final decision to terminate the trial	N/A
Harms	22	Plans for collecting, assessing, reporting, and managing solicited and spontaneously reported adverse events and other unintended effects of trial interventions or trial conduct	N/A
Auditing	23	Frequency and procedures for auditing trial conduct, if any, and whether the process will be independent from investigators and the sponsor	Page 8
**Ethics and dissemination**			
Research ethics approval	24	Plans for seeking research ethics committee/institutional review board (REC/IRB) approval	Page 10-11
Protocol amendments	25	Plans for communicating important protocol modifications (eg, changes to eligibility criteria, outcomes, analyses) to relevant parties (eg, investigators, REC/IRBs, trial participants, trial registries, journals, regulators)	Page 10-11
Consent or assent	26a	Who will obtain informed consent or assent from potential trial participants or authorised surrogates, and how (see Item 32)	Page 10-11
	26b	Additional consent provisions for collection and use of participant data and biological specimens in ancillary studies, if applicable	n/a

For peer review only - http://bmjopen.bmj.com/site/about/guidelines.xhtml

Confidentiality	27	How personal information about potential and enrolled participants will be collected, shared, and maintained in order to protect confidentiality before, during, and after the trial	Page 10-11
Declaration of interests	28	Financial and other competing interests for principal investigators for the overall trial and each study site	Page 12
Access to data	29	Statement of who will have access to the final trial dataset, and disclosure of contractual agreements that limit such access for investigators	Page 13
Ancillary and post-trial care	30	Provisions, if any, for ancillary and post-trial care, and for compensation to those who suffer harm from trial participation	N/A
Dissemination policy	31a	Plans for investigators and sponsor to communicate trial results to participants, healthcare professionals, the public, and other relevant groups (eg, via publication, reporting in results databases, or other data sharing arrangements), including any publication restrictions	Page 11
	31b	Authorship eligibility guidelines and any intended use of professional writers	-
	31c	Plans, if any, for granting public access to the full protocol, participant-level dataset, and statistical code	-

Appendices

For peer review only - http://bmjopen.bmj.com/site/about/guidelines.xhtml

Informed consent materials	32	Model consent form and other related documentation given to participants and authorised surrogates
Biological specimens	33	Plans for collection, laboratory evaluation, and storage of biological specimens for genetic or molecular analysis in the current trial and for future use in ancillary studies, if applicable

*It is strongly recommended that this checklist be read in conjunction with the SPIRIT 2013 Explanation & Elaboration for important clarification on the items. Amendments to the protocol should be tracked and dated. The SPIRIT checklist is copyrighted by the SPIRIT Group under the Creative Commons "Attribution-NonCommercial-NoDerivs 3.0 Unported" license.

For peer review only

BMJ Open

Use of a GP-endorsed 12 months' reminder letter to promote uptake of bowel scope screening: Protocol for a randomised controlled trial in a hard-to-reach population.

Journal:	*BMJ Open*
Manuscript ID	bmjopen-2018-022263.R1
Article Type:	Protocol
Date Submitted by the Author:	19-Apr-2018

Complete List of Authors:	von Wagner, Christian; UCL, Research Department of Behavioural Science and Health<br>Hirst, Yasemin; University College London, Research Department of Behavioural Science and Health<br>Tookey, Sara; UCL, Research Department of Behavioural Science and Health<br>Kerrison, Robert; University College London, Research Department of Behavioural Science and Health<br>Marshall, Sarah; London North West Healthcare NHS Trust, St Mark's Bowel Cancer Screening Centre<br>Prentice, Andrew; London North West Healthcare NHS Trust, St Mark's Bowel Cancer Screening Centre<br>Vulkan, Daniel; Queen Mary University of London, Policy Research Unit in Cancer Awareness, Screening and Early Diagnosis, Wolfson Institute of Preventive Medicine, Barts and The London School of Medicine and Dentistry<br>Macleod, Una; Hull York Medical School<br>duffy, stephen; Queen Mary University of London, 3Policy Research Unit in Cancer Awareness, Screening and Early Diagnosis, Wolfson Institute of Preventive Medicine, Barts and The London School of Medicine and Dentistry

**Primary Subject Heading**:	Public health
Secondary Subject Heading:	Gastroenterology and hepatology
Keywords:	Colorectal Cancer, Flexible Sigmoidoscopy, GP endorsement, Reminders

SCHOLARONE™
Manuscripts

Use of a GP-endorsed 12 months' reminder letter to promote uptake of bowel scope screening: Protocol for a randomised controlled trial in a hard-to-reach population.

Running Title: GP-endorsed reminders for bowel scope screening

Authors: von Wagner, Christian [1]; Hirst, Yasemin, Tookey, Sara [1]; Kerrison, Robert [1]; Marshall, Sarah[2]; Prentice, Andrew [2]; Vulkan, Daniel [3]; MacLeod, Una [4]; Stephen Duffy[3]

Affiliations: [1]Research Department of Behavioural Science and Health, University College London, Gower Place, London, UK

[2]St Mark's Bowel Cancer Screening Centre, London North West Healthcare NHS Trust, Harrow, United Kingdom

[3]Policy Research Unit in Cancer Awareness, Screening and Early Diagnosis, Wolfson Institute of Preventive Medicine, Barts and The London School of Medicine and Dentistry, Queen Mary University of London, London, UK

[4]Centre for Health and Population Sciences, Hull York Medical School, University of York, Heslington, York, UK

Corresponding Author: Dr Christian von Wagner, Research Department of Behavioural Science and Health, University College London, London WC1E6BT, UK; E-mail: c.wagner@ucl.ac.uk; Phone: +44 (0)20 7679 1614

Abstract word count: 296 ; **Main text word count:**;2699 **Figures:** 1 **Tables:** 0; Appendices: A, B

Keywords: Colorectal Cancer Screening; Flexible Sigmoidoscopy, GP-endorsement, reminders, London.

Version 1.6 19/04/2018

For peer review only

Abstract

Introduction: Flexible sigmoidoscopy (FS) screening is associated with reduced CRC incidence and mortality when offered as a one-off test to men and women aged 55-64. The test, also referred to as the 'bowel scope screening' (BSS) test, was added to England's national Bowel Cancer Screening Programme (BCSP) in March 2013, where it is offered to men and women aged 55. Since its implementation, uptake of the BSS test has been low, with only 43% of the eligible population attending an appointment. Sending non-participants a reminder at age 56 has been shown to improve uptake by up to nine percentage points at a single centre in London; we hypothesise that adding a General Practitioners (GPs) endorsement to the reminder could improve uptake even further.

Methods and analysis: This paper describes the protocol for a randomised controlled trial, which will examine the effectiveness of adding a GPs endorsement to a reminder for BSS non-participants aged 56. All screening-eligible adults who have not responded to a BSS appointment at London North West Healthcare NHS Trust within 12 months' of their initial invitation will be randomised to receive either a GP-endorsed reminder letter or reminder letter without GP endorsement. The primary outcome will be the proportion of individuals screened within each group eight weeks after the reminder. Statistical comparisons will be made using univariate and multivariate logistic regression, with 'uptake' as the outcome variable, GP reminder group as the exposure and socio-demographic variables as covariates.

Ethics and dissemination: The study was approved by the Yorkshire & Humber - Bradford Leeds Research Ethics Committee (16/YH/0298) and the Confidentiality Advisory Group (17/CAG/0162). The results will be disseminated in a peer-reviewed journal in accordance with the Consort statement and will be made available to the public.

Trial Registration: Current Controlled Trials ISRCTN 82867861

For peer review only - http://bmjopen.bmj.com/site/about/guidelines.xhtml

Strengths and limitations of the study

- If effective, this study will provide a cost-effective means to promote uptake of FS screening in an organised programme.
- The key importance of this randomised controlled trial protocol is that it highlights a methodology for delivering GP endorsed reminders to examine whether a 12 months' reminder letter can be further enhanced to improve screening uptake in an organised FS-based screening programme for CRC.
- There is a strong public health mandate to develop system friendly interventions to promote uptake of the BSS programme in England, particularly among socioeconomically deprived groups, where the potential for improving uptake is greatest.
- The study population is limited to the patients who are registered at the participating GP practices in London Boroughs of Hillingdon, Harrow and Brent.

For peer review only

For peer review only - http://bmjopen.bmj.com/site/about/guidelines.xhtml

Introduction

Colorectal cancer (CRC) is the fourth most common cancer in the United Kingdom and the second leading cause of cancer deaths [1; 2]. Screening is able to improve survival by detecting CRC early, when it is more treatable [3; 4; 5; 6]. Screening can additionally prevent cases through the early detection and removal of adenomas: the precancerous lesions from which most CRCs develop [7; 8; 9].

Randomised controlled trials (RCTs) examining the effectiveness of flexible sigmoidoscopy (FS) screening to improve CRC outcomes highlight that disease-specific mortality and incidence are reduced by 32% and 50% (respectively) when the test is performed just once between the ages of 55 and 64 [8; 10; 11; 12]. On this basis, the National Health Service (NHS) in England added 'once-only' FS screening for men and women aged 55 (known as Bowel Scope Screening, BSS) to the National Bowel Cancer Screening Programme (BCSP) in March 2013 [13]. Since that time, uptake of FS in the English BCSP has been only 43% [14], which is considerably lower than the uptake for the faecal occult blood test, which is offered biennially to those aged 60-74, and dramatically lower than rates observed for breast and cervical screening (54%, 76%, 78% respectively, [15; 16; 17]).

Barriers to FS screening include practical barriers (i.e., inconvenient appointment time, difficulties travelling to the appointment and so on), worry about pain, discomfort, or injury associated with the examination, and not wanting to know about any health issues [18]. Several studies attempting to address these barriers have examined the use of self-referral reminders (reminders which prompt former non-participants to self-refer for screening) 12 and 24 months after their initial invitation [19-21]. These have been shown to facilitate uptake in as much as 21.5% of former non-participants. While these results are highly promising, the annual reminder concept is still relatively unexplored, and there is considerable scope for modification and refinement of the intervention content. For example, there is now considerable evidence that a General Practitioners (GPs) endorsement of

For peer review only - http://bmjopen.bmj.com/site/about/guidelines.xhtml

cancer screening is positively associated with uptake [22- 24.]. Previous research examining the relative importance of barriers to CRC screening indicate that primary care should play a key role in encouraging uptake, highlighting that the two most important barriers to CRC screening among individuals who have never been screened are ‘failure of the clinician to suggest screening’ and ‘not knowing testing was necessary’ [25].

A recent study conducted in Australia demonstrated that a theory-based modification to the advanced notification letter improved uptake among men and was highly cost-effective [22]. A recent review has found that the inclusion of a GP’s endorsement on the invitation letter can improve uptake of CRC screening with the faecal occult blood test (FOBt); however, none of the studies identified in the review used flexible sigmoidoscopy screening uptake as an outcome [26]. This paper describes the protocol for a RCT which will examine the effectiveness of adding a GP endorsement to a reminder sent to BSS non-responders 12 months after their initial invitation.

For peer review only

Aims

The primary aim of this RCT will be to test whether adding a GP endorsement to the 12 months’ reminder letter improves the uptake of BSS among previous non-responders over and above a 12 months reminder letter without a GP endorsement. The secondary aim will be to examine demographic differences in uptake in response to the GP-endorsed reminder letter and the standard annual reminder letter (i.e. a reminder without a GP endorsement).

Methods and analysis

Study Design

This study will be a non-clinical RCT with two parallel arms (See Figure 1). The intervention group will receive a GP-endorsed reminder letter (Appendix A) by post, the control group will receive the same reminder letter by post, minus the GP-endorsement (Appendix B).

For peer review only - http://bmjopen.bmj.com/site/about/guidelines.xhtml

Study Setting

This RCT will be conducted in London, England, at London North West Healthcare NHS Trust in Harrow in between April and July, 2018.

Eligibility Criteria

Adults will be eligible to take part in the study if they: 1) are aged 56 years at the time of enrolment, 2) are registered with a consenting general practice served by the BSS centre at London North West Healthcare NHS Trust ; 3) have been offered, but not responded to a routine BSS appointment for at least twelve months; 4) meet the clinical eligibility criteria for BSS and 5) have not opted out from of sharing their personal data for purposes beyond direct care (from here on referred to as Type 2 objectors).

NHS Digital, formerly known as the Health and Social Care Information Centre, will be responsible for identifying potentially eligible adults and excluding any individuals who do not meet the criteria for the study. NHS Digital will exclude individuals if they: 1) have an open episode status, e.g. postponed the appointment to another date; 2) contacted the screening centre and declined; 3) contacted the screening programme and were deemed medically unfit; 4) contacted the screening programme to confirm they would be attending their appointment, but then did not attend.

Blinding & Randomisation

Adults who are eligible for inclusion will be entered into a computerised study database and randomised (in a 1:1 ratio) to receive either a ‘standard reminder letter’ or a ‘GP-endorsed reminder letter’ 12 months’ after their initial invitation using simple pseudo-random allocation methods.

Pre-intervention Phase: Recruitment of Practices

For peer review only - http://bmjopen.bmj.com/site/about/guidelines.xhtml

We will send invitations to GP practices which are being served by London North West Healthcare NHS Trust and have been included in the BSS programme for at least 12 months. Consenting GP practices will be permitting their practice name to be included in the GP-endorsed reminder letter. Using a recruitment strategy similar to a recent randomised controlled trial with GP practices in London, we are expecting approximately 50% of GP practices to sign up using a strategy of email invitations and weekly reminders [27].

Intervention Phase: Reminder delivery

The 12 months' reminder letter (Standard Reminder Group)

The standard reminder letter will be the same reminder letter that was used in the previous trials (see Appendix A ; [19-21]). It will be a personally addressed letter from BSS centre at the North West London Hospitals Trust that will invite recipients to make an appointment by returning an 'appointment-request-slip' or calling the Freephone number at the screening centre. As with the previous trials, the reminder will also give recipients the option to express a preference for the day and time of their appointment.

The 12 months' reminder letter with GP-Endorsement (Intervention Group)

The GP-endorsed reminder letter will be the same as the standard reminder letter, except that it will contain an additional statement of GP-endorsement, which states: "Your GP practice, xxxx xxxxx, supports this NHS Bowel Scope Screening Programme" (see Appendix B).

- Figure 1 Here –

Timeline

Twelve months after receiving an initial invitation for BSS, all eligible adults will receive a reminder letter (either the endorsed or standard letter) with the option of indicating preferred days and times to schedule their BSS appointment at the screening centre based at London North West Healthcare NHS Trust.

For peer review only - http://bmjopen.bmj.com/site/about/guidelines.xhtml

Sample size calculation

The study has been designed to detect a 5 percentage point increase in uptake between the GP-endorsed and standard reminder group. As a GP-endorsed reminder has not previously been tested in the context of an organised FS screening programme for CRC, the estimates for this study were based on the effect size of using GP-endorsement to promote uptake of screening for CRC using alternative tests (such as the faecal occult blood test: [22-24]). Hewitson and colleagues' [24] found that the response rate of individuals receiving usual care, versus a GP endorsed invitation letter, increased the rate of response by 5%, from 10% (as observed for a standard reminder in previous work) to 15%. To detect this increase in uptake with 80% power at the 5% alpha level, with two sided testing, approximately 600 participants per trial arm are required, giving a total sample size of 1,200 participants.

Data processing and data collection

1) Identification: Adults will be identified by NHS Digital only if they are registered with a consenting GP practice. Identification of eligible participants will take place using data contained within the Bowel Cancer Screening System (BCSS), which provides an up-to-date electronic record of uptake data for individuals enrolled in the national BSS.
2) Data Cleanse: NHS Digital will query the data of non-participants to identify type-II opt-outs. Using the cleansed dataset, the first 1200 individuals will be selected to be included in the RCT database.
3) Randomisation: NHS Digital will randomise eligible participants using the pre-randomised dataset. The database will be split into two separate files with an equal number of people in each document using the study groups.
4) Intervention: NHS Digital will share the data associated with each mail-out for the intervention and the control group with the mailing company called CFH Docmail Limited. CFH Docmail is an NHS Information Governance toolkit accredited mailing company that will be facilitating the mail merge throughout the study. The dataset will

For peer review only - http://bmjopen.bmj.com/site/about/guidelines.xhtml

include study group, unique ID, personal contact details, NHS number and the practice name (only for intervention group). Reminders will be produced by merging each study group database with the reminder letter templates. The full package will include: the reminder letter, standard information booklet and a freepost return envelope. This process of sending previous non-participants a reminder 12 months' after their initial will continue until the study sample size (n=1,200) is reached. We plan to send out 150 letters per week and send out all the reminders in eight weeks.

5) Data Generation: Once letters are sent, we will allow eight weeks for all individual's respond to the self-referral reminder. At the 16th week of the trial, NHS Digital will query the screening episode status of all individuals using their NHS numbers. If previous non-responders did not respond, their status will not change and they will remain 'non-responders'. If people confirmed and attended their appointment, they will be coded as 'attended'. If people contacted the centre and cancelled their appointment and did not book another appointment, they will be considered 'decliners'. If someone confirmed an appointment but did not attend, they will be coded as 'non-attenders'. We will also request a separate category for those who have contacted the screening centre, had the initial confirmation and attended the clinic but not eligible to have the full investigation. People will be adequately screened, if they have had the full bowel scope investigation and received a definitive clinical result. For this purpose, we will additionally request the screening outcome of those who had the screening, and also information about the follow-up colonoscopy investigations. As a result, we will be able to distinguish between: 1) those who reacted to the self-referral reminders (responders) vs. those who did not (non-responders), 2) those who successfully attended screening (attenders) vs. those who did not (non-attenders), and 3) those who were adequately screened vs. those who were not. In addition, NHS Digital will provide the Index of Multiple Deprivation (IMD) scores, using the postcodes of each individual included in the study.

For peer review only - http://bmjopen.bmj.com/site/about/guidelines.xhtml

6) Data Extraction: At the end of the study, after all data has been collated, the study database will be duplicated by NHS Digital. One copy will be anonymised for analysis by the UCL research team. In the anonymisation process, all identifiable information including NHS number, full name, Address, GP code, and GP name will be excluded.
7) Data Deletion: Once the anonymised dataset is sent to the research team at UCL and verified, the research team will ask CFH Docmail to destroy all the datasets that were shared by NHS Digital. Only the research team at UCL will have access to the final dataset.

Primary outcome

The primary outcome of this study will be the proportion of individuals attending a BSS appointment within each group. Uptake will be determined by checking the episode status of each individual included in the study 8 weeks after the distribution of the final reminder letter.

Statistical Analysis

The primary outcome will be analysed to test for a significant difference in uptake between the two groups using univariate and multivariate logistic regression, with 'adequately screened' as the outcome measure, 'trial arm' as the exposure and 'gender' and IMD as the covariates. Secondary analyses will look at whether there are differences in sociodemographic characteristics of non-responders, decliners and non-attenders. Sample characteristics will be reported using descriptive statistics. The comparison of overall uptake between trial arms will be presented using odds ratios (ORs) and 95% confidence intervals (CIs).

Ethics and dissemination

The timeliness and the feasibility of the study was approved by the Public Health England Bowel Cancer Screening Programme Research Advisory Committee (ID_192). The ethical considerations were reviewed by the Yorkshire & Humber - Bradford Leeds Research Ethics

For peer review only - http://bmjopen.bmj.com/site/about/guidelines.xhtml

For peer review only

Committee (16/YH/0298), who provided a favourable opinion. The study was also submitted to the Confidentiality Advisory Group (17/CAG/0162) for Section 251 exemption of the National Health Service Act 2006, which permits individual data to be processed without consent when the reasons for no consent is justified and the proposed study is in the public interest. However, the project website is designed to inform members of the public who will be included in the trial, timelines, objectives and how their data will be processed (http://www.ucl.ac.uk/iehc/research/behavioural-science-health/research/cancer-communication-screening/gp-reminders-for-bss-non-participants). As part of this patient and public notification, individuals are given the opportunity to opt-out in line with the CAG requirements. The explicit consent was not deemed necessary because the annual reminders are part being sent as routine practice at the BSS centre at London North West Healthcare NHS Trust as part of a Commissioning for Quality and Innovation (CQUIN) awarded by NHS England in 2017, and at the end of the project, the research team at UCL will not be receiving identifiable information. The exemption was necessary for the secure and fair data processing between NHS Digital and CFH Docmail. A Section 251 exemption was permitted to the CFH Docmail to receive the name, address, and the NHS number of the non-responders of BSS from NHS Digital for the duration of the study and subsequently post the annual reminders.

We will disseminate the outcomes of the study using academic publications, the study website, social media and also send a report to the GP practices that were invited to the study once the results are published. We will aim to publish the results in a peer-reviewed journal in accordance with the CONSORT statement. We will also use the study website to inform the public about the study outcomes: http://www.ucl.ac.uk/iehc/research/behavioural-science-health/research/cancer-communication-screening/gp-reminders-for-bss-non-participants.

Patient and Public Involvement

For peer review only - http://bmjopen.bmj.com/site/about/guidelines.xhtml

Members of the public were involved in the design of the self-referral reminder letter used in this study and those conducted before it [19, 20, 21]. Information on the study website, and the opt-out form were evaluated and informed by a patient representative. To make our findings more accessible to the public, we plan to report the outcomes of the trial on the study website in lay terms after the results are published in a peer reviewed journal.

Trial Registration

This trial has been registered with the International Standard Randomised Controlled Trial Number Registry (ISRCTN: 82867861).

Contributors

CVW and RK conceived of the study. ST, YH, CVW and RK participated in its design. ST, YH, CVW, RK and ST wrote the first draft of the manuscript. RK and YH participated in the power calculations and statistical analysis. DV, AP, SM, SD, UM were involved in revisions of the manuscript. All authors read and approved the final manuscript.

Acknowledgements

We would like to acknowledge Josephine Ruwende and Jean-Pierre Laake for their advice and assistance in the recruitment of GPs for this study. We thank Lindy Berkman, our patient representative, for her support in the development of the information provided to the public through the study website and included in the opt-out forms.

Funding

This work is part of the programme of the Policy Research Unit in Cancer Awareness, Screening, and Early Diagnosis, which receives funding for a research programme from the Department of Health Policy Research Programme (106/001). The funder has played play no role in study design and will have no influence in data collection, management, analysis

For peer review only - http://bmjopen.bmj.com/site/about/guidelines.xhtml

and interpretation or any decisions about whether and where to publish the results of the study.

It is a collaboration between researchers from seven institutions (Queen Mary University of London, University College London, King's College London, London School of Hygiene and Tropical Medicine, Hull York Medical School, Durham University and Peninsula Medical School).

For peer review only

Competing interests

The authors declare that they have no competing interests.

Provenance and peer review

Not commissioned; externally peer reviewed

Data sharing statement

The data that support the findings of this study are available from the Bowel Cancer Research Committee, but restrictions apply to the availability of these data, which were used under license for the current study, and so are not publicly available. Only the research team at UCL will have access to the final dataset. Anonymised data are can be made available from the authors upon reasonable request and with permission of the Bowel Cancer Research Advisory Committee after an application is made for to the Office for Data Release for a data sharing agreement.

For peer review only - http://bmjopen.bmj.com/site/about/guidelines.xhtml

References:

1. Cancer Research UK: Bowel Cancer Incidence Statistics. 2016a. from: http://www.cancerresearchuk.org/health-professional/cancer-statistics/statistics-by-cancer-type/bowel-cancer/incidence. (Accessed 22 Sept 2016).

2. Cancer Research UK: Bowel Cancer Mortality Statistics. 2016b. From: http://www.cancerresearchuk.org/health-professional/cancer-statistics/statistics-by-cancer-type/bowel-cancer/mortality. (Accessed 22 Sept 2016.)

3. Cancer Research UK: 2016c. from: http://www.cancerresearchuk.org/health-professional/cancer-statistics/statistics-by-cancer-type/bowel-cancer/survival. (Accessed 22 Sept 2016).

4. Sung J, Ng S, Chan F, Chiu H, Kim H, Matsuda T, Ng S, Lau J, Zheng S, Adler S. An updated Asia Pacific Consensus Recommendations on colorectal cancer screening. *Gut* 2015, 64(1):121-132.

5. Council of the European Union: Council Recommendation of 2 December 2003 on cancer screening. *Off J Eur Union* 2003; 878/EC: 34–38.

6. Logan RF, Patnick J, Nickerson C, Coleman L, Rutter MD, von Wagner C: Outcomes of the Bowel Cancer Screening Programme (BCSP) in England after the first 1 million tests. *Gut* 2012, 61(10):1439-1446. doi: 10.1136/gutjnl-2011-300843

7. Leslie A, Carey FA, Pratt NR, Steele RJC. The colorectal adenoma–carcinoma sequence. *Br J Surg* 2002, 89(7): 845-860. doi: 10.1046/j.1365-2168.2002.02120.x

For peer review only - http://bmjopen.bmj.com/site/about/guidelines.xhtml

8. Atkin WS, Edwards R, Kralj-Hans I, Wooldrage K, Hart AR, Northover JM, Parkin DM, Wardle J, Duffy SW, Cuzick J. Once-only flexible sigmoidoscopy screening in prevention of colorectal cancer: a multicentre randomised controlled trial. *Lancet* 2010, 375(9726): 1624-1633. doi: 10.1016/S0140-6736(10)60551-X.

9. Whyte S, Chilcott J, Halloran S. Reappraisal of the options for colorectal cancer screening in England. *Colorectal Dis* 2012, 14(9): e547-e561. doi: 10.1111/j.1463-1318.2012.03014.x

10. Hoff Geir, Grotmol Tom, Skovlund Eva, Bretthauer Michael. Risk of colorectal cancer seven years after flexible sigmoidoscopy screening: randomised controlled trial. *BMJ* 2009, 338 :b1846. doi: https://doi.org/10.1136/bmj.b1846

11. Schoen R., Pinsky P., Weissfeld J., Yokochi L., Church T., Laiyemo A., et al. Colorectal-cancer incidence and mortality with screening flexible sigmoidoscopy. *N Engl J Med*, 2012, 366: 2345-2357. doi: 10.1056/NEJMoa1114635

12. Segnan N., Armaroli P., Bonelli L., Risio M., Sciallero S., Zappa M., et al. Once-only sigmoidoscopy in colorectal cancer screening: follow-up findings of the Italian Randomized Controlled Trial–SCORE.2011. *J Natl Cancer Inst* 2001, 103: 1310–1322. doi: 10.1093/jnci/djr284

13. Department of Health NHS bowel cancer screening programme: Piloting of flexible sigmoidoscopy (FS) – Advice to the NHS and bidding process. 2012. from: https://www.gov.uk/government/publications/piloting-of-flexible-sigmoidoscopy-advice-to-the-nhs-and-bidding-procress (Accessed 23 Sept 2015).

For peer review only - http://bmjopen.bmj.com/site/about/guidelines.xhtml

14. McGregor LM, Bonello B, Kerrison RS, Nickerson C, Baio G, Berkman L, Rees CJ, Atkin W, Wardle J, von Wagner C. Uptake of Bowel Scope (Flexible Sigmoidoscopy) Screening in the English National Programme: the first 14 months'. *J Med Screen* 2015; e-pub ahead of print 20 September 2015. doi: 10.1177/0969141315604659

15. Von Wagner C, Baio G, Raine R, Snowball J, Morris S, Atkin W, et al. Inequalities in participation in an organized national colorectal cancer screening programme: results from the first 2.6 million invitations in England. *Int J Epidemiol* 2011;40(3):712–8. doi: 10.1093/ije/dyr008

16. Health & Social Care Information Centre (HSCIC). Breast screening Programme, England – 2014-15 [NS]. 2016. From: http://content.digital.nhs.uk/catalogue/PUB20018. (Accessed 10 Oct 2016).

17. Health & Social Care Information Centre (HSCIC). Cervical screening Programme, England – 2014-15 [NS]. 2016. http://content.digital.nhs.uk/article/2021/Website-Search?productid=19239&q=cervical+screening&sort=Relevance&size=10&page=1&area=both#top. (Accessed 10 Oct 2016).

18. Vernon SW. Participation in colorectal cancer screening: a review. *J Natl Cancer Inst* 1997, 89(19): 1406–1422.

19. Kerrison RS, McGregor LM, Marshall S, Isitt J, Counsell N, Wardle J, von Wagner C. Use of a 12 months'' self-referral reminder to facilitate uptake of bowel scope (flexible sigmoidoscopy) screening in previous non-responders: a London-based feasibility study. *British journal of cancer,* 2016 114(7):751-758. doi: https://doi.org/10.1093/jnci/89.19.1406

For peer review only - http://bmjopen.bmj.com/site/about/guidelines.xhtml

20. Kerrison, RS, McGregor, LM, Marshal, S, Isitt, J, Counsell, N, Rees, CJ, von Wagner, C. Improving uptake of flexible sigmoidoscopy screening: a Randomised Controlled Trial of non-participant reminders in the English Screening Programme. *Endoscopy* 2017, 49, 39-43. doi: 10.1055/s-0042-118452

21. Kerrison, R. S., McGregor, L. M., Counsell, N., Marshall, S., Prentice, A., Isitt, J., Rees, C. J. and von Wagner, C. (2018) 'Use of Two Self-referral Reminders and a Theory-Based Leaflet to Increase the Uptake of Flexible Sigmoidoscopy in the English Bowel Scope Screening Program: Results From a Randomized Controlled Trial in London', *Annals of Behavioral Medicine*. doi: 10.1093/abm/kax068.

22. Zajac IT, Duncan AC, Flight I, Wittert GA, Cole SR, Young GP, Wilson CJ, Turnbull DA. Theory-based modifications of an advanced notification letter improves screening for bowel cancer in men: A randomised controlled trial. Social Science & Medicine. 2016;165:1-9. doi: 10.1016/j.socscimed.2016.06.036

23. Raine R, Duffy SW, Wardle J, Solmi F, Morris S, Howe R, Kralj-Hans I, Snowball J, Counsell N, Moss S, Hackshaw A. Impact of general practice endorsement on the social gradient in uptake in bowel cancer screening. British journal of cancer. 2016 Feb;114(3):321.

24. Hewitson P, Ward AM, Heneghan C, Halloran SP, Mant D. Primary care endorsement letter and a patient leaflet to improve participation in colorectal cancer screening: results of a factorial randomised trial. *Br J Cancer* 2011, 105(4): 475-480. doi: 10.1038/bjc.2011.255

25. Jones, R. M., Woolf, S. H., Cunningham, T. D., Johnson, R. E., Krist, A. H., Rothemich, S. F. and Vernon, S. W. (2010) 'The Relative Importance of Patient-

For peer review only - http://bmjopen.bmj.com/site/about/guidelines.xhtml

Reported Barriers to Colorectal Cancer Screening', *American Journal of Preventive Medicine*. Elsevier, 38(5), pp. 499–507. doi: 10.1016/J.AMEPRE.2010.01.020.

26. Senore C, Inadomi J, Segnan N, Bellisario C, Hassan C: Optimising colorectal cancer screening acceptance: a review. Gut 2015: gutjnl-2014-308081. doi: 10.1136/gutjnl-2014-308081.

27. Hirst, Y., Skrobanski, H., Kerrison, R. S., Kobayashi, L. C., Counsell, N., Djedovic, N., Ruwende, J., Stewart, M. and von Wagner, C. ; Text-message Reminders in Colorectal Cancer Screening (TRICCS): a randomised controlled trial', *British Journal of Cancer* 2017; 116(11):1408-1414. doi: 10.1038/bjc.2017.117.

For peer review only

For peer review only - http://bmjopen.bmj.com/site/about/guidelines.xhtml

Figure legends

Figure 1: GP-Endorsed Reminders Consort Flow diagram

For peer review only

For peer review only - http://bmjopen.bmj.com/site/about/guidelines.xhtml

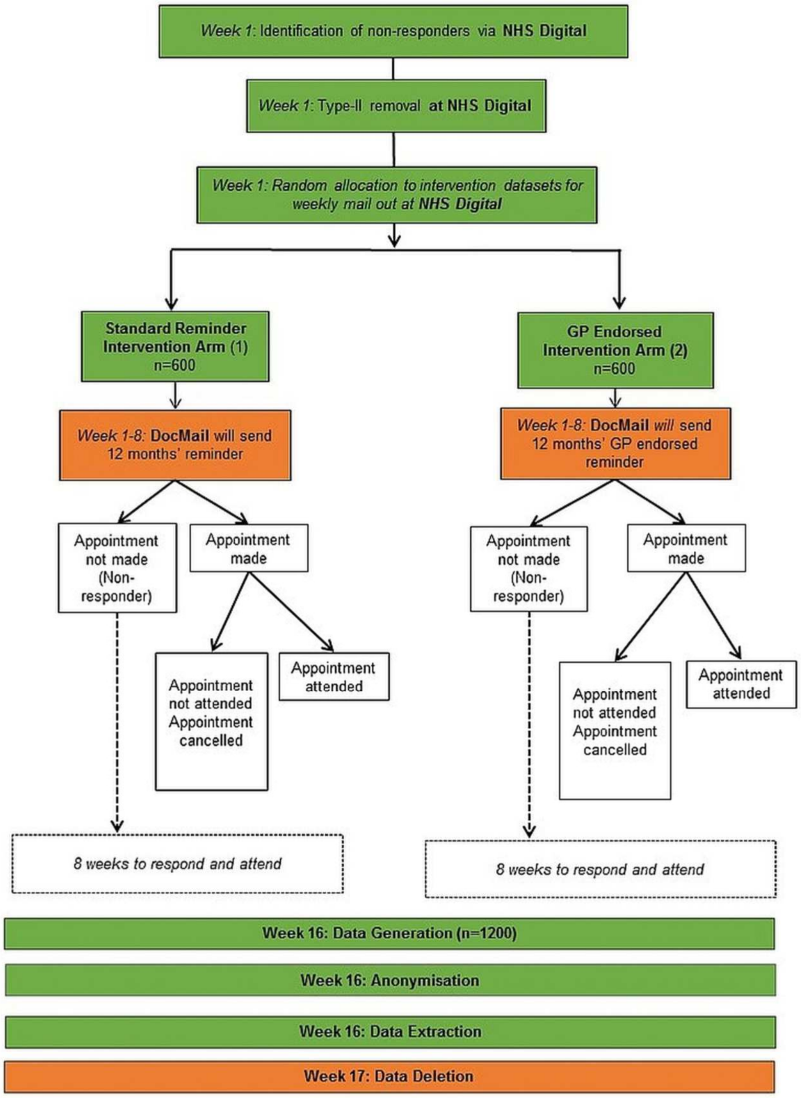

Figure 1

129x187mm (300 x 300 DPI)

The North West London Hospitals NHS Trust

St. Marks' Bowel Cancer Screening Centre
St Mark's Hospital
Watford Road
Harrow
Middlesex
HA1 3UJ

Freephone Helpline 0800 707 6060

<dd> <Month> <Year>

<Title> <First Name> <Last Name>
<Address Line 1>
<Address Line 2>
<Address Line 3>
<Address Line 4>
<Postcode>

Dear <Title> <First Name> <Initial> <Last Name>,
NHS No: <NHS Number>

Important information about your health:

We are writing to invite you for bowel scope screening, a new test available only in England that **helps prevent bowel cancer.** We last invited you for this test about a year ago.

People aged 55+ are most at risk of bowel cancer, this test helps prevent it:

We have written to you because people who are aged 55 and over are the most at risk of developing bowel cancer. Having a Bowel Scope Screening test between the age of 55 & 59 helps prevent you from getting bowel cancer in the future. This is an important test highly recommended for **everyone** who is **55-59** years of age.

Saving lives
The NHS offers bowel scope screening because it saves lives from bowel cancer.

Bowel scope screening is for people who **don't** have any signs of bowel cancer. The test is designed to **help prevent bowel cancer** by finding and removing small growths in the lower bowel before they turn into something more serious. These growths, called polyps, can turn into cancer over a period of years if they are left untreated. Removing these growths halves your risk of getting bowel cancer in the future.

We're lucky in Brent, Harrow, Hillingdon and Ealing that we have the opportunity to participate in bowel scope screening. Every month about 270 people take up the test.

What you need to do now
To book your test, simply fill in and post back the form overleaf in the Freepost envelope provided (you don't need a stamp).

We will then arrange a date and time for your bowel scope screening appointment. It takes place locally at St Mark's Hospital, which is a centre of excellence for bowel and gut medicine at Northwick Park.

Please read the enclosed leaflet, which gives more information about the test, and also has stories from people who have already been to St Mark's for bowel scope screening.

If you have any questions, please call the St Mark's Bowel Cancer Screening Centre on 020 8869 3543, or **Freephone 0800 707 60 60 to book an appointment**.

Yours sincerely,

Sarah Marshall
Clinical Programme Manager, Bowel Scope Screening

For peer review only - http://bmjopen.bmj.com/site/about/guidelines.xhtml

The North West London Hospitals NHS
NHS Trust

St Mark's Hospital Foundation
BEATING BOWEL DISEASE. SAVING LIVES.

IMPORTANT – PLEASE CHECK YOUR DETAILS AND RETURN IN THE FREEPOST ENVELOPE

Name: <Title> <First Name> <Last Name>
NHS Number: <NHS Number>
Post Code: <Postcode>

Please fill in your details (either your home telephone number or your mobile number is required; this is so we can contact you to confirm your appointment)**:**

Home number: ______________________

Mobile number: ______________________

(You may receive the details of your appointment in a text message reminder a week before your appointment)

Please **tick** this box if you would like to have a bowel scope screening appointment:

☐ I'd like to arrange a bowel scope screening appointment at St Mark's Hospital in Harrow.

Please **tick** as appropriate: My preferred appointment time(s) would be:

	**Afternoon 13:00-15:30**	**Evening 16:45-19:00**
**Monday**		
**Tuesday**		
**Wednesday**		
**Thursday**		
**Friday**		

When we receive your form, we'll contact you with a suggested date and time for your appointment.

(Tear here)

For peer review only - http://bmjopen.bmj.com/site/about/guidelines.xhtml

St. Marks' Bowel Cancer Screening Centre
St Mark's Hospital
Watford Road
Harrow
Middlesex
HA1 3UJ

Freephone Helpline 0800 707 6060

<dd> <Month> <Year>

<Title> <First Name> <Last Name>
<Address Line 1>
<Address Line 2>
<Address Line 3>
<Address Line 4>
<Postcode>

Dear <Title> <First Name> <Initial> <Last Name>,
NHS No: <NHS Number>

Your GP practice, WWWW WWWW WWWW WWWW WWWW WWWW WWWW WWWWW,
supports the NHS Bowel Scope Screening Programme

Important information about your health:

We are writing to invite you for bowel scope screening, a new test available only in England that **helps prevent bowel cancer.** We last invited you for this test about a year ago.

People aged 55+ are most at risk of bowel cancer, this test helps prevent it:

We have written to you because people who are aged 55 and over are the most at risk of developing bowel cancer. Having a Bowel Scope Screening test between the age of 55 & 59 helps prevent you from getting bowel cancer in the future. This is an important test highly recommended for **everyone** who is **55-59** years of age.

Saving lives

The NHS offers bowel scope screening because it saves lives from bowel cancer.

Bowel scope screening is for people who **don't** have any signs of bowel cancer. The test is designed to **help prevent bowel cancer** by finding and removing small growths in the lower bowel before they turn into something more serious. These growths, called polyps, can turn into cancer over a period of years if they are left untreated. Removing these growths halves your risk of getting bowel cancer in the future.

We're lucky in Brent, Harrow, Hillingdon, and Ealing that we have the opportunity to participate in bowel scope screening. Every month about 270 people take up the test.

What you need to do now

To book your test, simply fill in and post back the form overleaf in the Freepost envelope provided (you don't need a stamp).

We will then arrange a date and time for your bowel scope screening appointment. It takes place locally at St Mark's Hospital, which is a centre of excellence for bowel and gut medicine at Northwick Park.

Please read the enclosed leaflet, which gives more information about the test, and also has stories from people who have already been to St Mark's for bowel scope screening.

If you have any questions, please call the St Mark's Bowel Cancer Screening Centre on 020 8869 3543, or **Freephone 0800 707 60 60 to book an appointment**.

For peer review only

The North West London Hospitals NHS Trust

Yours sincerely,

Sarah Marshall
Clinical Programme Manager, Bowel Scope Screening

IMPORTANT – PLEASE CHECK YOUR DETAILS AND RETURN IN THE FREEPOST ENVELOPE

Name: <Title> <First Name> <Last Name>
NHS Number: <NHS Number>
Post Code: <Postcode>

Please fill in your details (either your home telephone number or your mobile number is required; this is so we can contact you to confirm your appointment)**:**

Home number: ________________________

Mobile number: _______________________

(You may receive the details of your appointment in a text message reminder a week before your appointment)

Please **tick** this box if you would like to have a bowel scope screening appointment:

☐ I'd like to arrange a bowel scope screening appointment at St Mark's Hospital in Harrow.

Please **tick** as appropriate: My preferred appointment time(s) would be:

	**Afternoon 13:00-15:30**	**Evening 16:45-19:00**
**Monday**		
**Tuesday**		
**Wednesday**		
**Thursday**		
**Friday**		

When we receive your form, we'll contact you with a suggested date and time for your appointment.

(Tear here)

GP endorsed 12 month's reminder letter, version 1.2, 18/07/2017, **IRAS ID: 200928** 2/2

For peer review only

STANDARD PROTOCOL ITEMS: RECOMMENDATIONS FOR INTERVENTIONAL TRIALS

SPIRIT 2013 Checklist: Recommended items to address in a clinical trial protocol and related documents*

Section/item	ItemNo	Description	Page Numbers
**Administrative information**			
Title	1	Descriptive title identifying the study design, population, interventions, and, if applicable, trial acronym	Page 1
Trial registration	2a	Trial identifier and registry name. If not yet registered, name of intended registry	Page 2
	2b	All items from the World Health Organization Trial Registration Data Set	N/A
Protocol version	3	Date and version identifier	Footer
Funding	4	Sources and types of financial, material, and other support	Page 12
Roles and responsibilities	5a	Names, affiliations, and roles of protocol contributors	Page 12
	5b	Name and contact information for the trial sponsor	Page 12.
	5c	Role of study sponsor and funders, if any, in study design; collection, management, analysis, and interpretation of data; writing of the report; and the decision to submit the report for publication, including whether they will have ultimate authority over any of these activities	Page 12

For peer review only

	5d	Composition, roles, and responsibilities of the coordinating centre, steering committee, endpoint adjudication committee, data management team, and other individuals or groups overseeing the trial, if applicable (see Item 21a for data monitoring committee)	Page 12
**Introduction**			
Background and rationale	6a	Description of research question and justification for undertaking the trial, including summary of relevant studies (published and unpublished) examining benefits and harms for each intervention	Page 4-5
	6b	Explanation for choice of comparators	
Objectives	7	Specific objectives or hypotheses	Page 5
Trial design	8	Description of trial design including type of trial (eg, parallel group, crossover, factorial, single group), allocation ratio, and framework (eg, superiority, equivalence, noninferiority, exploratory)	Page 6-10
**Methods: Participants, interventions, and outcomes**			
Study setting	9	Description of study settings (eg, community clinic, academic hospital) and list of countries where data will be collected. Reference to where list of study sites can be obtained	Page 6

For peer review only

Eligibility criteria	10	Inclusion and exclusion criteria for participants. If applicable, eligibility criteria for study centres and individuals who will perform the interventions (eg, surgeons, psychotherapists)	Page 6
Interventions	11a	Interventions for each group with sufficient detail to allow replication, including how and when they will be administered	Page 7
	11b	Criteria for discontinuing or modifying allocated interventions for a given trial participant (eg, drug dose change in response to harms, participant request, or improving/worsening disease)	N/A
	11c	Strategies to improve adherence to intervention protocols, and any procedures for monitoring adherence (eg, drug tablet return, laboratory tests)	N/A
	11d	Relevant concomitant care and interventions that are permitted or prohibited during the trial	N/A
Outcomes	12	Primary, secondary, and other outcomes, including the specific measurement variable (eg, systolic blood pressure), analysis metric (eg, change from baseline, final value, time to event), method of aggregation (eg, median, proportion), and time point for each outcome. Explanation of the clinical relevance of chosen efficacy and harm outcomes is strongly recommended	Page 10

For peer review only - http://bmjopen.bmj.com/site/about/guidelines.xhtml

Participant timeline	13	Time schedule of enrolment, interventions (including any run-ins and washouts), assessments, and visits for participants. A schematic diagram is highly recommended (see Figure)	Page 7
Page 7Sample size	14	Estimated number of participants needed to achieve study objectives and how it was determined, including clinical and statistical assumptions supporting any sample size calculations	Page 8
Recruitment	15	Strategies for achieving adequate participant enrolment to reach target sample size	Page8-9

Methods: Assignment of interventions (for controlled trials)

Allocation:

Sequence generation	16a	Method of generating the allocation sequence (eg, computer-generated random numbers), and list of any factors for stratification. To reduce predictability of a random sequence, details of any planned restriction (eg, blocking) should be provided in a separate document that is unavailable to those who enrol participants or assign interventions	Page 8
Allocation concealment mechanism	16b	Mechanism of implementing the allocation sequence (eg, central telephone; sequentially numbered, opaque, sealed envelopes), describing any steps to conceal the sequence until interventions are assigned	Page 8

Implementation	16c	Who will generate the allocation sequence, who will enrol participants, and who will assign participants to interventions	Page 8-9
Blinding (masking)	17a	Who will be blinded after assignment to interventions (eg, trial participants, care providers, outcome assessors, data analysts), and how	
	17b	If blinded, circumstances under which unblinding is permissible, and procedure for revealing a participant's allocated intervention during the trial	
**Methods: Data collection, management, and analysis**			
Data collection methods	18a	Plans for assessment and collection of outcome, baseline, and other trial data, including any related processes to promote data quality (eg, duplicate measurements, training of assessors) and a description of study instruments (eg, questionnaires, laboratory tests) along with their reliability and validity, if known. Reference to where data collection forms can be found, if not in the protocol	Page 8-10
	18b	Plans to promote participant retention and complete follow-up, including list of any outcome data to be collected for participants who discontinue or deviate from intervention protocols	

For peer review only - http://bmjopen.bmj.com/site/about/guidelines.xhtml

Data management	19	Plans for data entry, coding, security, and storage, including any related processes to promote data quality (eg, double data entry; range checks for data values). Reference to where details of data management procedures can be found, if not in the protocol	Page 8-10
Statistical methods	20a	Statistical methods for analysing primary and secondary outcomes. Reference to where other details of the statistical analysis plan can be found, if not in the protocol	Page 10
	20b	Methods for any additional analyses (eg, subgroup and adjusted analyses)	Page 10
	20c	Definition of analysis population relating to protocol non-adherence (eg, as randomised analysis), and any statistical methods to handle missing data (eg, multiple imputation)	N/A
**Methods: Monitoring**			
Data monitoring	21a	Composition of data monitoring committee (DMC); summary of its role and reporting structure; statement of whether it is independent from the sponsor and competing interests; and reference to where further details about its charter can be found, if not in the protocol. Alternatively, an explanation of why a DMC is not needed	N/A

For peer review only - http://bmjopen.bmj.com/site/about/guidelines.xhtml

	21b	Description of any interim analyses and stopping guidelines, including who will have access to these interim results and make the final decision to terminate the trial	N/A
Harms	22	Plans for collecting, assessing, reporting, and managing solicited and spontaneously reported adverse events and other unintended effects of trial interventions or trial conduct	N/A
Auditing	23	Frequency and procedures for auditing trial conduct, if any, and whether the process will be independent from investigators and the sponsor	Page 8
**Ethics and dissemination**			
Research ethics approval	24	Plans for seeking research ethics committee/institutional review board (REC/IRB) approval	Page 10-11
Protocol amendments	25	Plans for communicating important protocol modifications (eg, changes to eligibility criteria, outcomes, analyses) to relevant parties (eg, investigators, REC/IRBs, trial participants, trial registries, journals, regulators)	Page 10-11
Consent or assent	26a	Who will obtain informed consent or assent from potential trial participants or authorised surrogates, and how (see Item 32)	Page 10-11
	26b	Additional consent provisions for collection and use of participant data and biological specimens in ancillary studies, if applicable	n/a

For peer review only

Confidentiality	27	How personal information about potential and enrolled participants will be collected, shared, and maintained in order to protect confidentiality before, during, and after the trial	Page 10-11
Declaration of interests	28	Financial and other competing interests for principal investigators for the overall trial and each study site	Page 12
Access to data	29	Statement of who will have access to the final trial dataset, and disclosure of contractual agreements that limit such access for investigators	Page 13
Ancillary and post-trial care	30	Provisions, if any, for ancillary and post-trial care, and for compensation to those who suffer harm from trial participation	N/A
Dissemination policy	31a	Plans for investigators and sponsor to communicate trial results to participants, healthcare professionals, the public, and other relevant groups (eg, via publication, reporting in results databases, or other data sharing arrangements), including any publication restrictions	Page 11
	31b	Authorship eligibility guidelines and any intended use of professional writers	-
	31c	Plans, if any, for granting public access to the full protocol, participant-level dataset, and statistical code	-

Appendices

review only

For peer review only - http://bmjopen.bmj.com/site/about/guidelines.xhtml

Informed consent materials	32	Model consent form and other related documentation given to participants and authorised surrogates
Biological specimens	33	Plans for collection, laboratory evaluation, and storage of biological specimens for genetic or molecular analysis in the current trial and for future use in ancillary studies, if applicable

*It is strongly recommended that this checklist be read in conjunction with the SPIRIT 2013 Explanation & Elaboration for important clarification on the items. Amendments to the protocol should be tracked and dated. The SPIRIT checklist is copyrighted by the SPIRIT Group under the Creative Commons "Attribution-NonCommercial-NoDerivs 3.0 Unported" license.

For peer review only
